# Identification of small molecule inhibitors of Interleukin-18

**DOI:** 10.1038/s41598-017-00532-x

**Published:** 2017-03-28

**Authors:** Brian Krumm, Xiangzhi Meng, Yan Xiang, Junpeng Deng

**Affiliations:** 1 Department of Biochemistry and Molecular Biology, Oklahoma State University, Stillwater, OK 74078 USA; 2 Department of Microbiology, Immunology and Molecular Genetics, University of Texas Health Science Center at San Antonio, 7703 Floyd Curl Drive, San Antonio, TX 78229 USA; 3 The University of North Carolina, School of Medicine, Pharmacology, Chapel Hill, North Carolina, 27599 United States

## Abstract

Interleukin-18 (IL-18) is a pleiotropic pro-inflammatory cytokine belonging to the IL-1 superfamily. IL-18 plays an important role in host innate and adaptive immune defense but its aberrant activities are also associated with inflammatory diseases such as rheumatoid arthritis and Crohn's disease. IL-18 activity is modulated *in vivo* by its naturally occurring antagonist, IL-18 Binding Protein (IL-18BP). Recent crystal structures of human IL-18 (hIL-18) in complex with its antagonists or cognate receptor(s) have revealed a conserved binding interface on hIL-18. Through virtual screening of the National Cancer Institute Diversity Set II and *in vitro* competitive ELISA we have identified three compounds (NSC201631, NSC80734, and NSC61610) that disrupt hIL-18 binding to the ectromelia virus IL-18BP. Through cell-based bioassay, we show that NSC80734 inhibits IL-18-induced production of IFN-γ in a dose-dependent manner with an EC_50_ of ~250 nM. Our results and methodology presented here demonstrate the feasibility of developing small molecule inhibitors that specifically target the rather large interface of IL-18 that is involved in extensive protein-protein interactions with both IL-18BP and its cognate receptor(s). Our data therefore provide the basis for an approach by which small molecules can be identified that modulate IL-18 activity.

## Introduction

Interleukin-18 (IL-18) is a pleiotropic pro-inflammatory cytokine belonging to the IL-1 superfamily^1–3^. IL-18 plays an important regulatory role in both innate and acquired immune responses against pathogenic infections. IL-18 was originally referred to as IFN-γ Inducing Factor (IGIF) for its ability to stimulate the production of IFN-γ^3, 4^. IL-18 stimulates IFN-γ production from T-helper lymphocytes cells (Th1) and macrophages, and enhances the cytotoxicity of natural killer (NK) cells. The IL-18 stimulated IFN-γ production is synergistically amplified with other Th1-related cytokines, IL-2, IL-15, IL-12 and IL-23^5–8^. IL-18 is synthesized as a 23 kDa inactive precursor, which is subsequently cleaved into an 18 kDa active form by a member of the inflammasome (Interleukin-1β Converting Enzyme, ICE (Caspase-1)) and then secreted, resulting in the initiation of IL-18 signaling cascade^3, 9^. IL-18 signals through its two membrane bound receptors, IL-18Rα and IL-18Rβ, forming a ternary complex necessary for productive intracellular signaling^10^. IL-18 activity is modulated *in vivo* by Interleukin-18 Binding Protein (IL-18BP), a soluble protein comprised of a single Immunoglobulin (Ig) domain^11, 12^. The human IL-18BP (hIL-18BP) has an exceptionally high affinity for hIL-18 of 400 pM and has been shown to be up-regulated in various cell lines in response to elevated IFN-γ levels, suggesting that it serves as a negative feedback inhibitor of hIL-18 mediated immune response^12, 13^.

Despite its significant role in host immune response against infection, aberrant hIL-18 bioactivity has been associated with inflammatory and autoimmune diseases, allergies, and neurological disorders^8, 14, 15^. In fact, it has been shown that increased levels of mature hIL-18 have a direct correlation with the severity of pathological autoimmune diseases such as Multiple Sclerosis (MS), Rheumatoid Arthritis (RA), and lupus^16^. Therefore, down regulating hIL-18 bioactivity seems to be a logical approach for treatment of inflammatory and autoimmune diseases. A current strategy for treating these human diseases is to target proteins involved in the initiation event(s) of inflammation or upstream events of the innate immune response. These upstream effector proteins include but are not limited to Cyclooxygenase-2 (Cox-2) and Caspase-1, which respond to Non-Steroidal Anti-Inflammatory Drugs (NSAID) or specific caspase inhibitors, respectively. However these treatments suffer from side effects such as colitis^17^.

There exist potential therapies that involve the use of antibodies directed against the interface of hIL-18 and hIL-18Rα or the use of recombinant hIL-18BP, both of which are being tested in clinical trials^18, 19^. Recombinant hIL-18BP has been shown to be effective at treating inflammatory skin diseases and LPS-induced liver injury^20, 21^. The use of hIL-18BP to treat these pathological conditions has met with some success in clinical trials but has also met with complications often causing immunogenic reaction themselves^16^. Therefore, protein-based immunotherapy strategies face potential drawbacks such as immunogenic rejections, and other such complications especially with immune compromised individuals^16, 22, 23^. Developing small molecule inhibitors presents a novel approach for down regulating hIL-18 bioactivity in part due to their bioavailability and might also serve as better alternatives.

**Figure 1:**
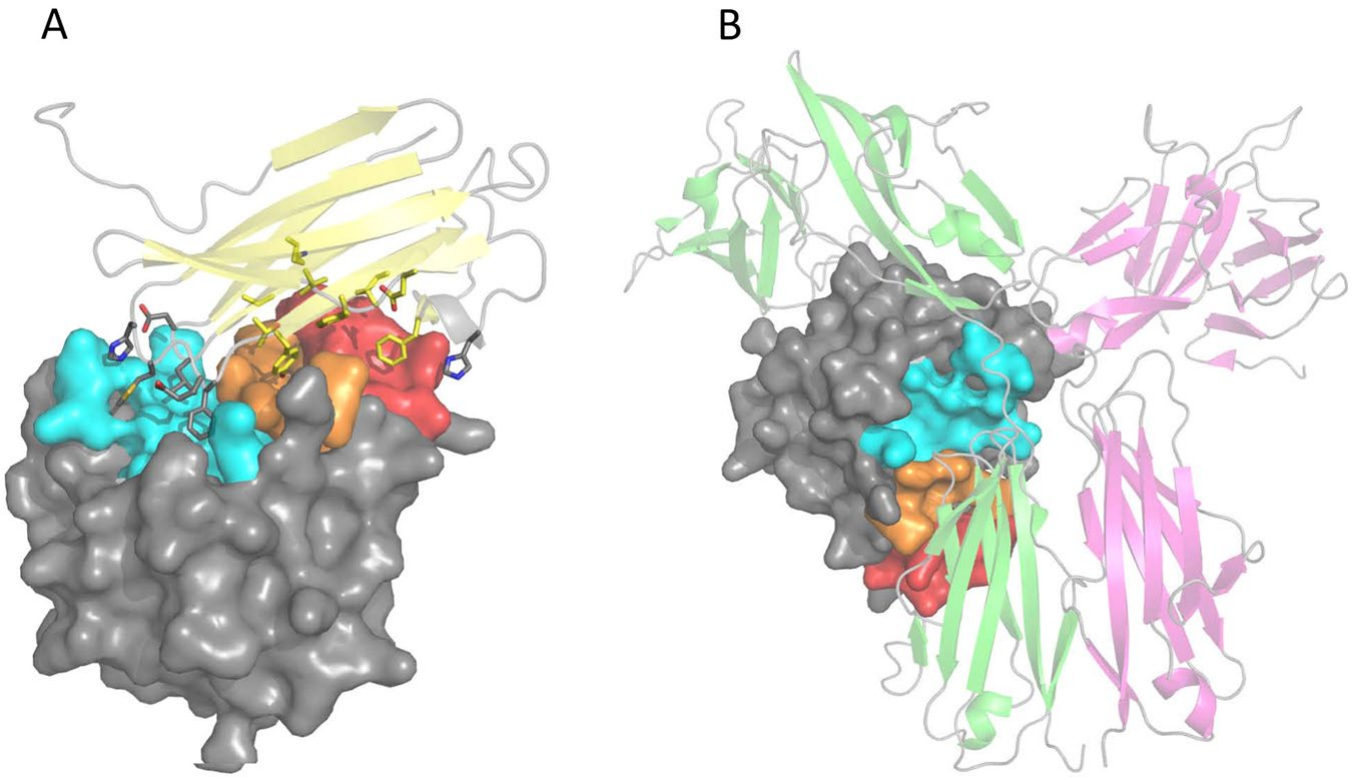
Human IL-18 Viral and Ternary Receptor Complex Crystal Structures. (**A**) Crystal structure of hIL-18:ectvIL-18BP viral complex, PDB entry 3F62. Depicted in ribbon diagram and colored in light yellow is ectvIL-18BP, hIL-18 is drawn in surface representation. Residues of ectvIL-18BP at the interface are shown as sticks, interacting with previously identified binding sites on hIL-18 colored as site A (red), site B (orange), and site C (cyan). The remaining surface of hIL-18 is colored in grey. (**B**) Crystal structure of hIL-18 ternary receptor complex, PDB entry 3WO4. Drawn as cartoon representation and colored green and magenta are hIL-18 Receptor α and β, respectively. Human IL-18 has been drawn as seen in panel A.

Functional IL-18BPs, natural IL-18 inhibitors, are not limited to just vertebrates but are also encoded by many poxviruses including Molluscum Contagiosum Virus (MCV) and orthopoxviruses. It has been shown that IL-18BP from poxviruses species of ectromelia and vaccinia virus contributes to virulence by down-modulating IL-18 mediated immune responses, suggesting a possible role as a decoy for human immune evasion^24, 25^. The molecular mechanism by which IL-18BP modulates hIL-18 signaling has been elucidated from two recent high-resolution crystal structures of hIL-18 in complex with two divergent IL-18BPs from ectromelia (ectv^26^), and yaba-like disease virus (yldv^27^). It was shown that both IL-18BPs bind to the same surface of hIL-18 used by other IL-18BPs, suggesting that all IL-18BPs, including hIL-18BP, use a conserved inhibitory mechanism by blocking a conserved surface on hIL-18 that is commonly shared for binding hIL-18Rα (Fig. 1). The crystal structures of hIL-18 in complex with viral IL-18BPs revealed that the interface on hIL-18 is hydrophobic in nature with as much as 1,930 Å^2^ buried surface area^26^ comprised of three distinct sites A, B, and C. This interface is also shared by hIL-18Rα D3 domain for signaling^10^. Contained within hIL-18 binding site A is a critical hIL-18 Lysine residue (K53) when mutated to alanine displayed a greater than 100- and 4-fold decrease in binding affinity for variola IL-18BP and hIL-18Rα, respectively^28^. In fact, K53A mutation of hIL18 increased IL-18 bioactivities due to its reduced ability of being inhibited by IL-18BP, indicating that binding site A on hIL-18 surface is a ‘hot spot’. Additionally, hIL-18 binding site C is also another potential ‘hot spot’ as it pockets a highly conserved orthopoxvirus Phenlyalanine that has been shown to be critical for orthopoxvirus, molluscum contagiosum virus (MCV), and human IL-18BP complex formation^27, 29–31^. Alanine substitutions of residues that surround binding site C, (G108, H109, and K112) abrogated hIL-18 ternary complex formation^10^. The identified surface patches on hIL-18 could therefore be targeted toward designing small molecule compounds as rational inhibitors against hIL-18 activity.

Protein-protein interactions are associated with most crucial biological processes including signal transduction, cell adhesion, cellular proliferation, growth and differentiation to name a few while their malfunctions have been identified in numerous pathological disease states^32^. It is estimated that up to 650,000 interactions regulate biological processes within the human cell with these protein-protein interactions having been termed the ‘interactome’^33^. While most small molecule drugs currently on the market are either competitive inhibitors of G-protein-coupled receptors, nuclear receptors, ion channels or an enzymatic target, there exist a small but increasing number of protein-protein inhibitors (PPIs) showing a degree of success in clinical trials^34–36^. Protein-protein inhibitor discovery focuses on targeting binding clefts and ‘hot spots’ at protein interfaces for which small molecules or fragments can modulate their activity. However, this is not always effective noting that protein-protein interfaces are very large ranging from 1,300–3,000 Å^2^ in buried surface area and tend to be on average hydrophobic in nature^37, 38^. To date, small molecule inhibitors of IL-2 exemplify rational drug design targeting cytokines. IL-2 is a cytokine with a four-helix bundle structure. A panel of small molecules that binds to IL-2 directly were developed at Sunesis Pharmaceuticals with the tightest binding molecule having a disassociation constant in the mid-nanomolar range. These small molecules bind directly on IL-2 and disrupt the interaction between IL-2 and the a-chain of the IL-2 receptor^39–41^.

To date, there are no published data for small molecules specifically targeting hIL-18 or its receptor complex. The identification of small molecules targeting hIL-18 could potentially open up new avenues for the discovery of novel therapeutics for the treatment of pathological diseases associated with hIL-18 bioactivity. Based on the structural data from hIL-18 and viral IL-18BPs complexes, we used Autodock^42^ to perform *in silico* screening of small molecule compounds representative of the National Cancer Institute (NCI) depository. The inhibitory effects of potential small molecule leads identified from *in silico* screening were confirmed by an *in vitro* competitive ELISA assay between hIL-18 and ectvIL-18BP. We also show in a cell-based assay that the identified small molecule inhibitors blocked hIL-18 stimulated IFN-γ production.

## Results

Using the Autodock program^43, 44^ we performed virtual screening for small molecules that could potentially fit into the identified IL-18 binding interface pockets. Approximately 1,500 compounds from the NCI Diversity Set II ligand library representing greater than 250,000 compounds in the NCI repository were used to identify potential inhibitors. We designed a grid box incorporating the hIL-18 surface (including site A, B, and C) for docking of compounds and searched for the lowest possible binding energy of potential inhibitors. Autodock returned predicted binding poses grouped in binding clusters with a root mean square deviation (r.m.s.d.) tolerance of less than 1 Å between poses of the cluster. The results were evaluated by ranking various complexes toward the predicted binding energy. Cluster analysis was subsequently accomplished on the basis of r.m.s.d. values with respect to the starting ligand geometry. The docked conformation with the most favorable binding free energy and the more populated cluster was selected as the best result. For each cluster, the estimated free energy of binding in kcal mol^−1^ was obtained and an estimated inhibition constant (Ki) at 298.15 K was derived. Virtual screening of the NCI Diversity Set II identified twenty-seven unique compounds with a predicted Ki of less than 1 uM, which were subsequently obtained from the NCI Developmental Therapeutics Program (NCI/DTP, http://dtp.nci.nih.gov).

**Figure 2:**
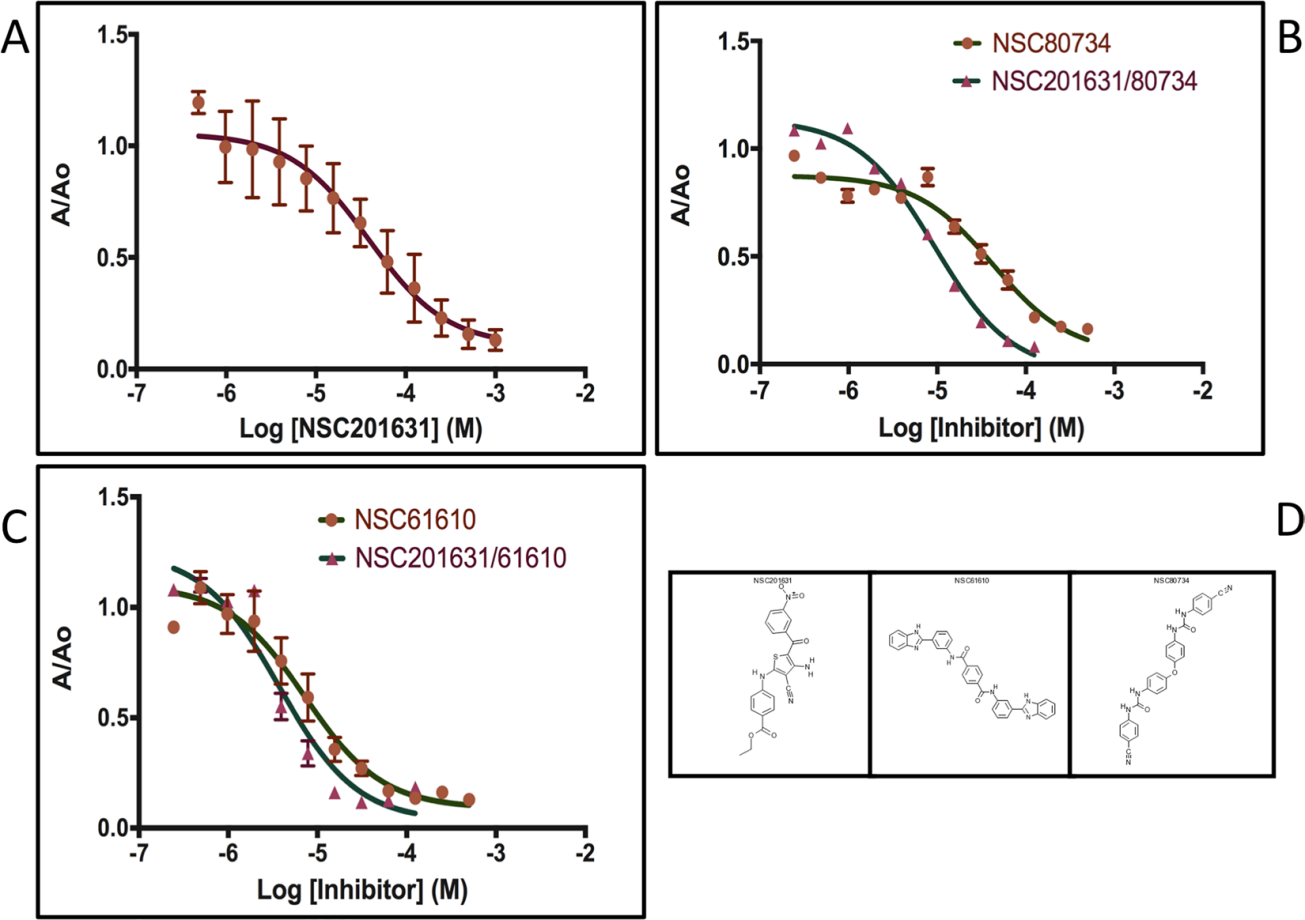
Competitive ELISA and IC_50_ determination. Dose-response curves with varying compound concentrations for the inhibition of hIL-18:ectvIL-18BP complex formation. IC_50_ values were obtained by nonlinear regression fitting to a variable slope, three-parameter dose-response model using the GraphPad Prism 6 software (San Diego, CA, USA). Competitive ELISA experiments were performed using four technical replicates. (**A**) Competitive ELISA curve for NSC201631, IC_50_ = 44+/− 9 uM, n = 3. (**B**) Competitive and Synergistic ELISA results for NSC80734, IC_50_ = 52 + / 38 uM, n = 3 and NSC201631/80734 (IC_50_ = 9.3 uM). (**C**) Competitive and Synergistic ELISA results for NSC61610, IC_50_ = 6+/− 5 uM, n = 2, and NSC201631/61610 (IC_50_ = 3.7 uM) (**D**) Two-Dimensional Chemical Structure of NSC201631, NSC61610, an NSC80734. Note: Synergistic competitive ELISA results obtained from four technical replicates of a single assay (n = 1). Standard deviation from mean is plotted with error bars.

We assessed the ability of the twenty-seven compounds for inhibiting the binding between hIL-18 and ectvIL-18BP by using a competitive ELISA method. Since the interface of hIL-18:IL-18BP is shared by IL-18Rα, ectvIL-18BP is used in the ELISA assays as an easy readout for screening inhibitors of IL-18 signaling. Specifically, we added biotinylated hIL-18 together with varying concentrations of the compound to the wells coated with purified MBP-ectvIL-18BP. We detected the amount of bound hIL-18 using a streptavidin-HRP conjugate in the presence of 5% (v/v) DMSO alone and a proven non-inhibitory compound were used as negative controls. Three compounds, NSC201631 (ethyl 4-[[4-amino-3-cyano-5-(3-nitrobenzoyl)thiophen-2-yl]amino]benzoate), NSC80734 (1-(4-cyanophenyl)-3-[4-[4-[(4-cyanophenyl)carbamoylamino]phenoxy]phenyl]urea), and NSC61610 (1-N,4-N-bis[3-(1H-benzimidazol-2-yl)phenyl]benzene-1,4-dicarboxamide) inhibited binding in a dose-dependent manner with IC_50_ of 44+/− 9 uM (n = 3), 52+/− 38 uM (n = 3) and 6+/− 5 uM (n = 2), with calculated Ki of 1.6 uM, 1.8 uM, and 0.2 uM, respectively (Fig. 2).

**Figure 3:**
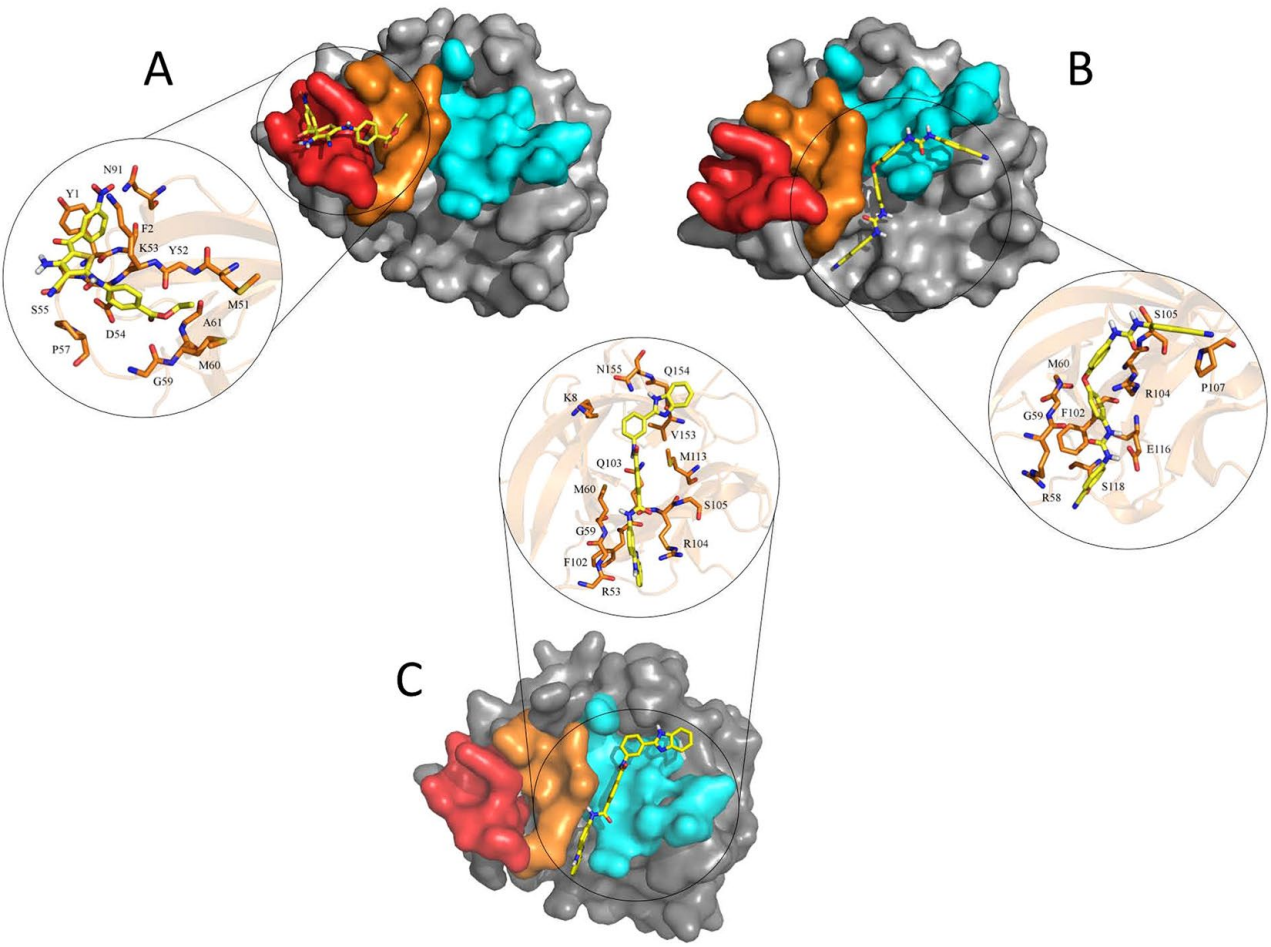
Virtual Docking of Identified Compounds. Autodock docking poses of compounds (**A**) NSC201631, (**B**) NSC80734, and (**C**) NSC61610. The docked conformation with the most favorable binding free energy and the most populated cluster was selected as the best result. Previously identified binding sites on hIL-18 colored as site **A** (red), site **B** (orange), and site **C** (cyan) with the remaining surface of hIL-18 is colored in grey. Each individual compound is colored in yellow and shown using a stick figure. Zoomed inserts for each figure illustrate the predicted interactions of the compound with hIL-18 residues. For clarity, non-interacting residue sidechains have been omitted. Compound NSC201631 docked with a predicted potential of 8 hydrogen bonds and 25 hydrophobic interactions; NSC80734 docked with a predicted potential of 8 hydrogen bonds and 24 hydrophobic interactions; NSC61610 docked with a predicted potential of 10 hydrogen bonds and 26 hydrophobic interactions.

In virtual screening, compound NSC201631 had a predicted Ki of 903 nM and docked into the induced fit cavities of binding sites A and B with a majority of the molecule residing in binding site A (Fig. 3A); NSC80734 had a predicted Ki of 409 nM and docked with half of the molecule laying off to the side of hIL-18 between β strands β5 and β8, the other half of the molecule docked on to the side of binding site C (Fig. 3B); NSC61610 had a predicted Ki of 88 nM and docked across the β-trefoil opening extending from one side of β-trefoil opening to the other and was anchored similar to NSC80734 with one end of the molecule inserted between β strands β5 and β8 and the other end interacting with the C-terminus β12 while occupying a significantly greater area of binding site C when compared to NSC80734 (Fig. 3C). The calculated Ki values derived from the competitive ELISA data were approximately 2-fold lower for NSC201631 and NSC61610 while approximately 4-fold lower for NSC80734 than the predicted Autodock Ki values. Thus, the ranking order of these compounds based on the Ki values was different for Autodock and the competitive ELISA but NSC61610 still had the highest inhibition constant for both.

**Figure 4:**
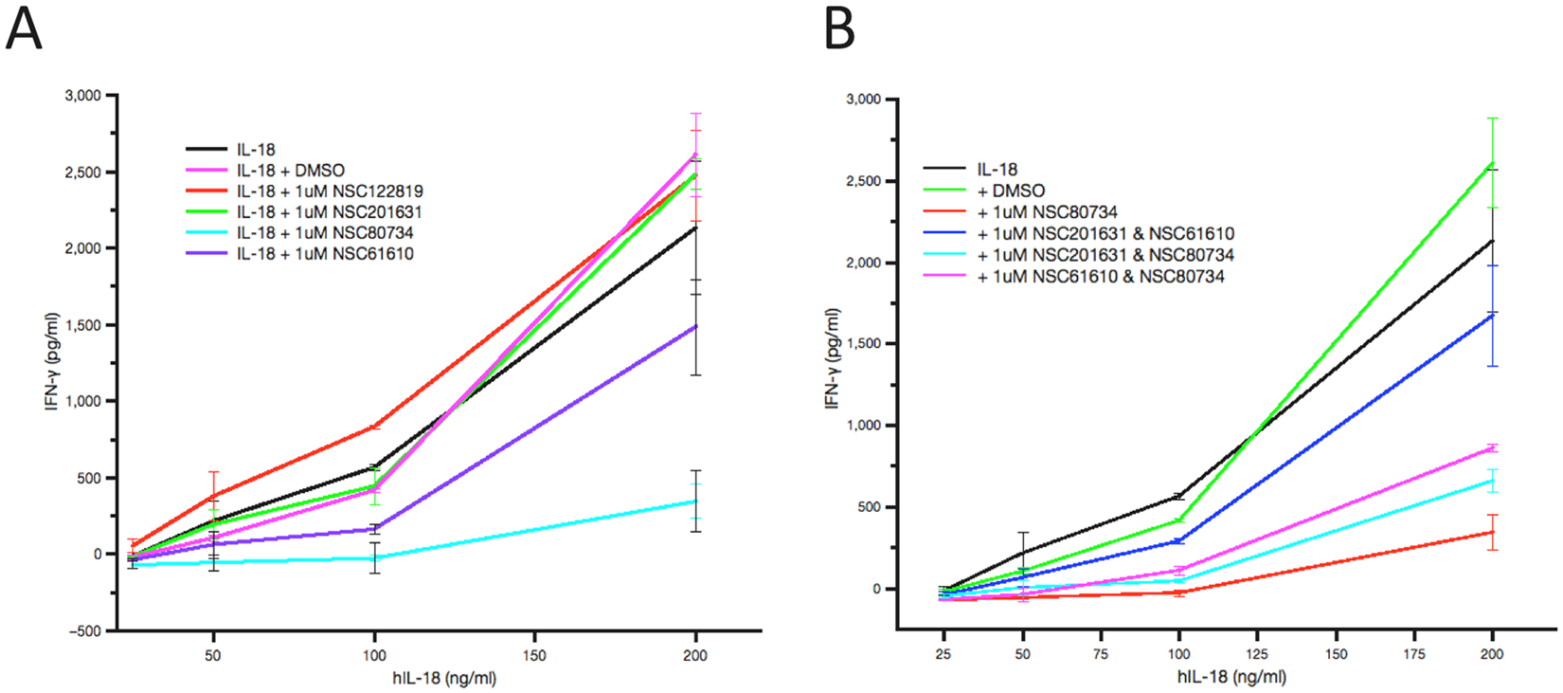
IFN-γ Secretion Assay with selected inhibitors. Human IL-18 bioassay for the detection of secreted IFN-γ using increasing concentrations of hIL-18. (**A**) Inhibition curves obtained with a constant 1 uM inhibitor concentration and a cell concentration of 1 × 10^6^ cells ml^−1^. (**B**) Synergistic inhibition curves obtained by adding a final concentration of 1 uM total inhibitor of both compounds, cell concentration was 1 × 10^6^ cells ml^−1^.

**Figure 5:**
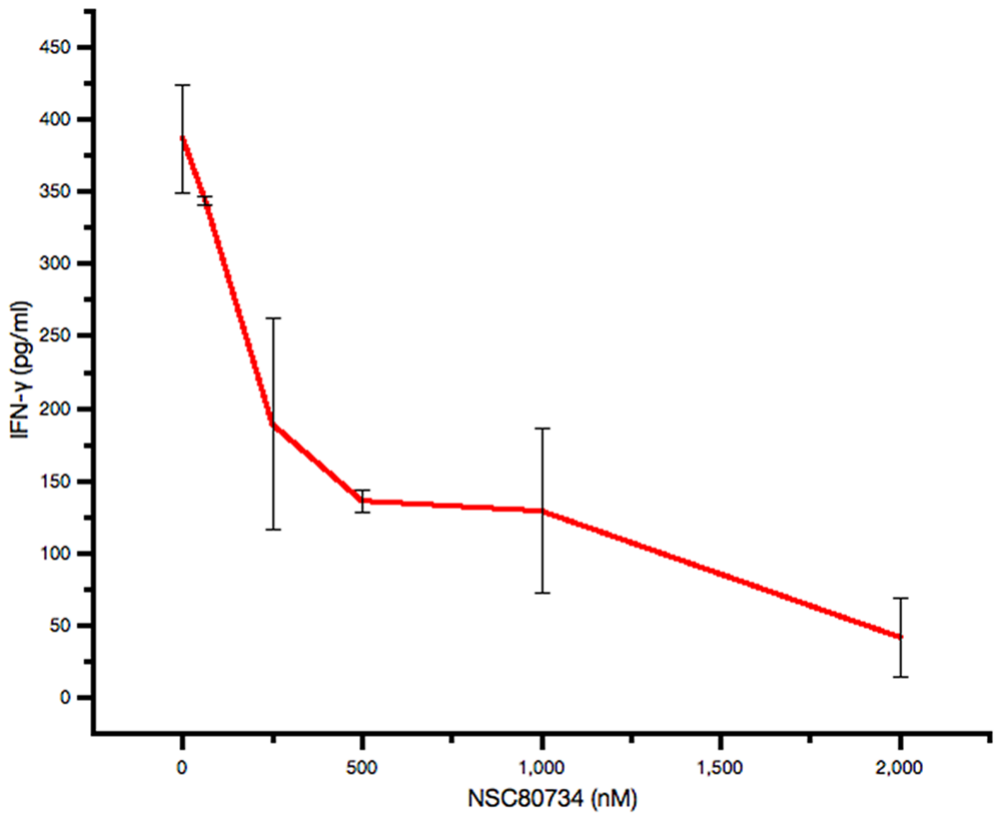
Dose dependent inhibition of IFN-γ Production. Human IL-18 bioassay for the detection of secreted IFN-γ at a constant concentration of hIL-18. Inhibition curves obtained with increasing concentration of NSC80734 and a cell concentration of 1 × 10^6^ cells ml^−1^.

As the three identified compounds docked in different surface pockets on hIL-18 and compounds NSC80734 and NSC61610 docked in different poses in binding site C, we wanted to determine if there exist any synergistic or additive effects between these three compounds. Combining NSC201631 and NSC80734 had the most significant additive effect with an approximate 5- to 6-fold increase in IC_50_ in the competitive ELISA with an IC_50_ of 9.3 uM (n = 1) (Fig. 2B) when comparing the individual compounds suggesting they occupy two non-overlapping sites on hIL-18 and consistent with the predicted docking pose(s). Combining NSC201631 and NSC61610 had a modest additive effect with a slight increase in IC_50_ of 3.7 uM (n = 1) (Fig. 2C) when compared to NSC61610 alone but also consistent with occupying two non-overlapping binding sites. Human IL-18 induces IFN-γ production in human KG-1 cells. We used this bioassay and tested the three compounds for their ability to inhibit hIL-18 activities. Interestingly, NSC201631 did not inhibit hIL-18 activities, while NSC61610 had a small inhibitory effect on hIL-18 activities. Compound NSC80734 displayed the best inhibition of hIL-18 induced stimulation of IFN-γ in a dose-dependent manner with an EC_50_ of ~250 nM (Figs 4 and 5).

## Discussion

Protein-protein inhibitor discovery focuses on targeting binding clefts and ‘hot spots’ at protein interfaces for which small molecules or fragments can modulate their activity. However, this is not always effective noting that protein-protein interfaces are very large ranging from 1,300–3,000 Å^2^ in buried surface area and tend to be on average hydrophobic in nature^37, 38^.

The crystal structures of hIL-18 in complex with viral IL-18BPs revealed that the interface is hydrophobic in nature with as much as 1,930 Å^2^ buried surface area^26^. This interface is comprised of three distinct sites A, B, and C, which are also shared by hIL-18Rα D3 domain for signaling. Human IL-18 binding site A and B cavities were occupied by ectvIL-18BP residues Y51, Y53, and F67 in the complex structure, these residues are highly conserved in poxvirus species and play critical roles (Y53 and F67) in binding hIL-18^28–31^. Mutations to residues surrounding hIL-18 binding site C completely eliminated ternary complex formation^10^. Human IL-18 surface sites A, B, and C are thus appropriately sized for small-molecule binding.

Our docking and competitive ELISA data suggest that compounds NSC201631 and NSC61610 would bind to the surface of hIL-18 and thus prevent viral and human IL-18BPs complex formation. Interestingly, although compound NSC80734 showed less binding to hIL-18 when compared to NSC61610 (Fig. 2B and C) in our competitive ELISA experiment, it displayed the best inhibition on IL-18 signaling in our cell-based bioassay (Fig. 4A and B). It is speculative that NSC80734, upon binding to site C on IL18, could disrupt the recruitment of hIL-18Rβ into the signaling receptor ternary complex preventing downstream hIL-18 mediated signaling. The data also suggest the surface on IL-18, although shared between IL-18BP and IL-18 receptors, may have varying energetic contributions to the binding of either protein. Thus the discovery of these three compounds (NSC201631, NSC80734, and NSC61610) through virtual screening, competitive ELISA, and bioassay serves as a proof-of-principle. The data provides a unique opportunity for structure based drug design to develop more potent inhibitors that could discriminate between hIL-18BP and hIL-18 ternary receptor complex formation for the treatment of not only diseases attributed to orthopoxvirus infections but also to pathogenic human diseases attributed to the aberrant expression of hIL-18. Further detailed structure-activity studies on these inhibitors will be needed for development of compounds with tighter binding affinities, improved efficacy and perhaps toxicity. All of which should benefit and be guided by high-resolution structures and medicinal chemistry.

## Experimental Procedures

### Virtual Screening

*In silico* docking of hIL-18 with potential inhibitors was carried out using the Autodock program (version 4.2) from the Molecular Graphic Laboratory of the Scripps Research Institute^43, 45^. AutoDock uses a genetic algorithm to generate the poses of the ligand inside a known or predicted binding site utilizing the Lamarckian version of genetic algorithm where the conformational changes of molecules after *in situ* optimization are used as subsequent poses for the offspring. Since there are no known structures of hIL-18 bound with inhibitors reported to date, we used a quasi “blind” docking method for defining the potential inhibitor binding locations on the surface of hIL-18^46^. In this approach, the entire hIL-18 interface that was involved in binding viral IL-18BP was used in docking to screen for potential inhibitors with lowest possible binding energy. The crystal structure of hIL-18 in complex with ectvIL-18BP (PDB entry 3F62) was used for our docking experiments^26^. Apo hIL-18 was generated by editing the pdb file and removing the ectvIL-18BP and all water molecules from the complex structure. Gasteiger charges were placed on the structure of apo hIL-18 using tools from the Autodock suite. The induced fit hIL-18 interface was used for docking purposes and the ligands were considered to be flexible. A grid box for ligand docking experiments was centered on the hIL-18 β-trefoil opening using 0.375 Å spacing with x, y, and z coordinates of 5.3, 10.2, 12.0 and a definition of 102 Å × 106 Å × 58 Å points, respectively. Additional experimental parameters used in Autodock include setting the population size to 300, the number of generations to 27,000 and the number of evaluations to 20,000,000. The number of docking runs was set to 50 with a cutoff of 1 Å for the root mean square deviation (r.m.s.d.) for grouping of each docking run. As a starting point for virtual screening, the NCI Diversity Set II (http://dtp.cancer.gov) ligand library was used for docking experiments. The Diversity Set II contains approximately 1,500 compounds representing the greater than 250,000 compounds of the NCI repository in a structural-data file (sdf) format^47^. For use in the Autodock program, the Diversity Set II sdf format was converted to three-dimensional coordinates at pH 7.4 in mol2 (Sybyl) format using the program Open Babel^48^. Gasteiger charges were generated for each compound using the scripts provided by the Autodock suite.

### Protein Expression and Purification

The coding sequence for mature ectvIL-18BP (residues 21–126) was cloned into a modified pET28b expression vector as a fusion protein with an N-terminal 6x histidine tagged maltose binding protein (MBP) moiety. The coding sequence of tobacco etch virus (TEV) protease recognition site (ENLYFQG) was constructed in frame between MBP and ectvIL-18BP. This vector was transformed into *E. coli BL21 (DE3)* Rosetta-Gami 2 cells (Invitrogen) and grown in 2xYT media until A_600_ reached 0.8–1.0, at which time the temperature was lowered to 18° C. Protein expression was induced with a final concentration of 0.5 mM IPTG and allowed to continue for an additional 18 hrs. The MBP-ectvIL-18BP fusion protein was purified using the similar procedure as described^49^. Briefly, the fusion protein was purified first from the soluble cell lysates using a Ni-NTA affinity column. The MBP-ectvIL-18BP was further purified through size exclusion chromatography with a Superdex 200 column, concentrated to 4 mg ml^−1^, then flash frozen and stored at −80° C until usage^50^. Mature human IL-18 was cloned into a modified pET vector containing a C-terminal 6x His tag along with the coding sequence for a biotinylation sequence tag (GLNDIFEAQKIEWHE). The plasmids of IL-18 and biotin ligase (Cat # AVB101, www.avidity.com) were used for co-transformation into *E. coli BL21 gold (DE3)* cells. The biotinylated IL-18 was expressed and purified similarly as described above.

### Competitive Enzyme-Linked Immunosorbant Assay (ELISA)

We developed a chromogenic competitive ELISA that displays reduced signal output when complex formation is perturbed between hIL-18 and ectvIL-18BP. Briefly, 50 ul of 1 ug ml^−1^ of MBP-ectvIL-18BP in 50 mM carbonate buffer, pH 9.6 was coated to Nunc Immulon Maxisorp plates (Thermo Scientific) at 37° C for 90 min. Plates were washed with a 1X PBS (50 mM Sodium Phosphate, 150 mM Sodium Chloride, pH 7.4) solution and then blocked for 3 hr at room temperature with a 1% (w/v) casein solution (Sigma Aldrich) in 1X PBS solution. Plates were air dried at room temperature and then stored desiccated at 4° C until the assay was performed. The inhibitors were provided from NCI and made to stocks at 10–20 mM concentration in 100% DMSO (Sigma Aldrich). The inhibitor stocks were subsequently diluted into 100% DMSO until an appropriate final inhibitor concentration was achieved. Purified biotinylated hIL-18 protein was diluted to 1 ug ml^−1^ then rapidly mixed with the inhibitor dilutions such that a final concentration of 5% (v/v) DMSO was achieved. 50 ul of the IL-18/inhibitor mix was added to each well and incubated at room temperature for 1 hr. Plates were washed 3 times with 1X PBST (PBS + 0.05% (v/v) Tween-20) and subsequently incubated with 50 ul of a 1:1000 dilution of a 1 mg ml^−1^ Streptavidin-HRP (Horse Radish Peroxidase, Thermo Scientific) conjugate in 1X PBS for 1 hr at room temperature. Plates were washed again and developed with 50 ul of 3,3′,5,5′-tetramethylbenzidine (TMB) solution (Pierce) for 30 min at room temperature. The assay was stopped with 150 ul of 2 N sulphuric acid (Sigma Aldrich) and the absorbance at 450 nm was measured using a VersaMax microplate reader (Molecular Devices). Least squares fit analysis and IC_50_ were determined using GraphPad Prism 6 software (San Diego, CA, USA). Inhibition constant (Ki) were calculated with MBP-ectvIL-18BP, hIL-18 uM concentrations and a disassociation constant (K_d_) of 0.2 nM using the web-based server BotDB^51^.

### IFN-γ BioAssay

The hIL-18 bioassay was done similarly as previously described^52^. Briefly, KG-1 cells (0.25 ml at 1 × 10^6^ cells ml^−1^) in RPMI medium containing 10% (v/v) Fetal bovine Serum were seeded in wells of a 96-well plate and stimulated with different concentrations of hIL-18. After stimulation at 37 °C for 24 hr, 100 ul of clarified supernatants was assayed for the secretion of human IFN-γ via ELISA (BD Biosciences), each hIL-18 assay was done in duplicates (n = 2).
